# From absolute nonsense to the world’s operating system

**DOI:** 10.1007/s12525-021-00508-w

**Published:** 2021-10-31

**Authors:** Hannes Werthner

**Affiliations:** TU Wien, Favoritenstrasse 9, Vienna, Austria

**Keywords:** Digital humanism, Digital transformation, Data ownership, D60, I31, L10

## Abstract

Informatics, its artefacts and methods, have fundamentally changed our society and world, from the individual personal level up to the current geo-political powerplay between the US, China and Europe. Information Technology serves as the operating system of our society. This change has happened over the last 30 – 40 years, and the result should be compared with our historic views and expectations. At the same time, despite the enormous success of our discipline, it has serious shortcomings. I discuss some of them, especially the issue of online platforms, and describe a positive “answer”: Digital Humanism. This approach does not only describe and analyze the man – machine relationship, but also underlines the importance of humans in this development: we have to interfere – technology is not god given. This fundamental idea is already supported by a growing number of academics and practitioners from different disciplines and fields.

## Introduction

“This is absolute nonsense” was the response by the audience, I remember, at the first ENTER conference on IT and tourism, in Innsbruck in 1994. Beat Schmid from the University of St. Gallen in Switzerland, founder of the Competence Center on Electronic Markets (CCEM), gave a talk about electronic markets, and Larry Press (from the University of California in Los Angeles foresaw and even described digital agents as copies of ourselves in the digital world. The majority of the audience – both scientific as well as industrial – was really skeptical. However, many of those engaged in the organization of that conference in 1994 are today Professors (some already retired) and / or successful entrepreneurs.

I start this contribution with the reference to the at that time utopian views on our future, where even we had our doubts. The comparison of this historical anecdote with today’s situation, with nearly all these expectations having become true, or even being excelled, provides the background of my personal view on the short history of our discipline. Today we see that IT, or Informatics and its artefacts and methods, is a major driver of change in industry and society, from the individual personal level up to the geo-political powerplay between US, China and Europe. However, this transformation comes with some shortcomings, which I will shortly discuss. These need an answer like the Vienna Manifesto on Digital Humanism, which is the core of my contribution. I finish with a short, rather subjective, look into the future. We are in a critical situation, which calls for our attention and our response, as academics as well as citizens. This is my conviction, being engaged in the field since nearly 40 years, and having provided substantial contributions, especially in the field of e-tourism.

Today we experience – and are astonished by its transformative power – the complex, technical socio-economic process called Digital Transformation. This is now at the surface, where 35 years ago this was called nonsense. This transformation has also changed our understanding of Informatics and IT from a mere engineering discipline dealing with a »stand-alone« computer, to a worldwide endeavor that touches on every aspect of our lives. The current global pandemic has only further underscored and demonstrated IT’s role as the »Operating System« of our society. As stated by (Lee, 2020), we experience the co-evolution of man – machine.

In the following I use the term “Informatics” and not Computer Science (see also (Tedre & Denning, 2015) for a discussion of the numerous, mainly artefact focused definitions of Computer Science). They also highlight the changing “semantics” of the term Computer Science over time, from a tool to a method of science. I also distinguish Informatics from the discipline Information Systems, which also looks at the organizational settings and embeddings of IT systems. Informatics is broader with respect to both, it does not deal with the computer and its use alone, but as defined by Turing Award winner Kristen Nygaard “Informatics is the science that has as its domain information processes and related phenomena in artifacts, society and nature” (Nygaard, 1986) Following this definition, Informatics has a holistic view on these developments, taking also into consideration its broader impact. This introduces a social and political dimension and the necessary need to look beyond algorithmic optimization, efficiency, or even profitability.

## The system is failing

IT systems are useful and extremely successful – see the current pandemic crisis; without our tools we would have a complete lock down. IT does not only keep the system running, it also serves for solving fundamental and vital problems (e.g., the essential role of informatics with respect to the Sustainable Development Goals (SDG) of the UNO^1^). This example, however, also shows that Informatics alone does not suffice, big problems need an interdisciplinary approach, integrating several disciplines. At the same time this development comes with downsides, as stated already in 2018 by Tim Berners-Lee in (The Guardian, 12.03.2018) »The system is failing«. Others even excused themselves for what they did (e.g., Jaron Lanier, an early Silicon Valley virtual reality pioneer in an interview in the New York Magazine, 16.04.2018)^2^. Also, Vardi (2019) in »How the Hippies Destroyed the Internet« describes very well that the original idea of the freedom of information – information as a free public good - led ultimately, and following the logic of the *tragedy of the commons* (Hardin, 1968), to an advertising-driven business model and to monopolistic structures. And there are other critical issues:

- The developments in AI and a*utomated decision-making* – put simply, the representation and automation of human thought – may result in autonomous decision-making systems, with substantial legal and ethical questions (Larus et al., 2018).
- Software components – as part of the technology stack - will increasingly control all machines (*every machine touched by SW becomes a computer*), this will have massive impact on employment and jobs – in both qualitative and quantitative terms. Productivity will continue to increase drastically and generate wealth; how will it be distributed?
- We see violations of privacy on a massive scale, both by private companies and by state instances (Zuboff, 2019). This development will require both legal and technical control measures. At the same time the World Wide Web makes terror attacks easier, as it has itself already become a medium for warfare.
- These developments are also evident in the political arena, e.g., in the intentional fabrication of fake news and the creation of opinion bubbles in the Web. The use of the Web for unfair political influence will lead to drastic political disputes, in particular with the focus on future political online decision-making processes.
- The role of IT has become so essential on economic, political and even military level that it is an issue of geo-political sovereignty. Take as an example the EU. It has, not being the home base of one single key-player in the IT platform economy, realized that it needs either to establish its own public IT infrastructure (see also (Kagermann & Wilhelm, 2020), and their European Public Sphere proposal) or to impose hard regulation rules on these systems, with all the related enforcement problems.

## Online platforms

“Electronic markets have become the commercial face of the global IT infrastructure”, already observed by Alt and Klein (2011), and much more as foreseen in the talk of Beat Schmid, mentioned at the beginning. Platforms are *the* dominant intermediation structure and economic environment, although they do not offer all the features of a full electronic market place – in fact, some of them focus only on specific subsets of transaction services. These platforms could also be characterized by a kind of dialectic relationship between cooperating networks and at the same time centralization around platform operators, which created and control these structures. However, they are the two sides of the same coin, in this economic environment network effects with its dynamics led to a situation where a small number of players dominate the market (Parker et al., 2016; Parker, 2019). They increase market efficiency through the reduction of transaction costs, as has already been discussed by (Williamson, 1985). From on organizational perspective it has been recently argued that platforms are a new form of an organizational model, in addition to hierarchies, markets and networks (Stark & Pais, 2021). The authors state that platforms show similarities to all three forms, but cannot be reduced to them.

In October 2020, when looking at market valuation, 7 of the top 10 companies were platform companies, with five coming from US and two from China. In some markets these companies have markets shares of 50 – 90 percent (e.g., search engine). Compare this with the year 2013 when only two of those companies were in the top 10. As shown by (Cusumano, 2019) these companies make their turnover only with half of the number of employees, and approx. double profit (comparing the largest 43 publicly listed platform companies to 100 of the largest firms in the same businesses over a 20-year period)^3^. The Corona crisis both reveals as well as it is at the same time intensifying this circumstance.

Focusing on transactional services the big platforms are industrial “sector” independent, as such they are orthogonal to industrial domains (you can buy “everything” on Amazon), the concrete product plays almost no role; it is virtualized, as are companies, entire markets and increasingly also our society. In addition, these platforms are innovation drivers (Gawer & Cusumano 2013), where we observe platform competition instead of product competition. For companies and organizations such dynamic networked environments call for network engineering instead of process reengineering (Werthner, 2001), important is not how efficient you perform, but what is the added value you can provide. This was already recognized by (Venkatraman, 1994) and his five-level framework of how IT may transform a business. It starts from a localized exploitation of IT (level one), with the intermediate level three of redesigning business processes, ending at level five with the redesign of the scope of a business. At this level IT enables an organization to redefine what it does (for others) and how to position itself in the related business network.

In this context the airline reservation systems (CRS / GDS) of the 60s and 70s may serve as historic examples (see Copeland and McKenney, 1988). Also in these cases computing power and technical competence led to new market structures and dominant market positions of few players. However, in contrast to the current situation, these systems were highly industry domain dependent, had no real plans to enter other industrial areas, and, maybe most important, they were created by “traditional” existing market players. The new players of today have a self-understanding as IT companies, are coming from outside, and diffusing into all economic and societal sectors, with new technical and market services. In these fast-changing circumstances, it is difficult, as already noted by Clemons and Madhani (2010), to define “their” sector and to address monopoly abuses of digital businesses by regulation. This observation is confirmed by the current regulation discussions, both in the EU as well as in the USA.

These multinational technology companies have power that national democratic governments have serious problems to control through traditional regulatory approaches. Our institutional frameworks are not able to deal with such a situation. In essence, those companies know more about citizens than the respective states, they offer services states do not and cannot provide with that quality, these companies decide – and not the states – on the implementations of crucial services (e.g., the case of the European Corona Apps– see the discussion on ““Corona Contact Tracing – the Role of Governments and Tech Giants“; https://dighum.ec.tuwien.ac.at/lectures-program/)^4^.

## The Vienna Manifesto

This specific “double-sided” role of IT, its indisputable enormous achievements and potential, and at the same time it’s now obvious critical issues, which are interlinked and connected, have led to the Vienna Workshop on Digital Humanism, in Vienna in April 2019. The intellectual point of departure was our responsibility as scientists (Popper, 1969), calling upon us to shape technologies according to human values and needs, instead of allowing technologies to shape humans. The workshop was also inspired by the tradition of the Vienna Circle, a multidisciplinary effort of the early twentieth century to reflect on the revolutionary implications of science, and Physics in particular, for our understanding of the world.

The term »Digital Humanism« was intentionally chosen to refer to the concepts of Humanism and Enlightenment, according to which the human is responsible for his or her own thinking and is at the focus (see Nida-Rümelin & Weidenfeld, 2018). We have the freedom, the right and the responsibility to make use of our own thought power, we are the authors of our own lives; personal autonomy and freedom to make decisions are the prerequisites for an open, democratic society. Technological progress is not a »divine gift«, we as individuals and as society should and must make decisions taking democratic and humanistic considerations into account. We define Digital Humanism as an approach that describes, analyzes, and, most importantly, influences the complex interplay of technology and humankind, for a better society and life, fully respecting universal human rights.

Over 100 participants from academia, public institutions, business and civil society took part in the two-day workshop. The program addressed the history and impacts of information technology, as well as the dynamics and future of the sector. The very lively discussions focused on technical, political, economic, social, ethical and legal issues. A real benefit was the presence of such a diversity of disciplines, covering political science, legal science, sociology, history, anthropology, philosophy, management science and informatics. The discussion showed that informatics alone – although important – is not enough to provide comprehensive answers; much more a broad, interdisciplinary approach is called for. The participants were also convinced that it is possible to influence these developments, indeed that it is our responsibility to do so Table 1.

**Table 1:** Principles of the Vienna Manifesto

**Privacy, democracy and inclusion** • Digital technologies should be designed in such a way that they promote democracy and inclusion. • Privacy and freedom of speech are basic values which should be at the center of our activities.	
**Regulation and public oversight** • The regulatory authorities must intervene to break up technology monopolies. • Decisions whose consequences could affect individual or collective human rights must still be made by humans.	
**Specific role of science and the academic sector** • Scientific approaches integrating various disciplines and eliminating discipline-specific silos are needed for mastering our challenges. • Universities are the places where new knowledge is created and critical thinking is exercised. They should eliminate the boundaries among disciplines and fostering their collaboration toward a holistic view of technological development.	
**Education and training** • New curricula are required which combine Humanities, Social Sciences and Technical and Engineering Sciences. • Education in IT and training work on the ethical and societal impacts of IT must begin as early as possible in the education process.	

At the workshop a »Vienna Manifesto for Digital Humanism«^5^ was discussed and adopted by signatories from nearly 50 countries as blueprint for shared principles. The Manifesto is primarily, however, a call to act collectively to mobilize support that transcends national borders and continents in order to build a more human and common future. More specifically, the “principles” of the Vienna Manifesto are shown in the following table:

## Changed landscape

We touched an obvious up-to-date and hot topic; the response was enormous. Not only academics from the Informatics fields reacted, but also from other disciplines, as was the expressed interest by the civil society, funding agencies and even political decision makers.^6^ In addition, there are a number of international initiatives with similar objectives with which we started to network, e.g., HAI - Human-Centered Artificial Intelligence at Stanford^7^, Center for Humane Technology^8^, Dutch Digital Society^9^, or The Digital Enlightenment Forum^10^. All these activities point into the same general direction of a human centered digital future, with, however, some differences with respect to the disciplinary breadth or specific technology focus (e.g., AI only). As explained in the previous chapter, our focus is on IT in general, and the insight that we need a broad disciplinary coverage as well as different types of activities from research up to policy making for having societal impact. In addition, our membership goes beyond Universities and academia, in difference to some of the other initiatives.^11^

The growing public awareness is also reflected by recent political actions; it shows that things are changing:

- In the US antitrust lawsuits against Facebook and Google.
- In December 2020 the Europe Commission announced the *Digital Service Act* and the *Digital Market Act*, focusing on the regulation of the online “world”.
- In December 2020 the responsible ministers of all EU member states signed the *Berlin Declaration on Digital Society and Value-Based Digital Government*. The declaration contains points raised in our Manifesto such as the promotion of fundamental rights and democratic values in the digital society.
- Or – on a different level – the Vienna government announced the foundation of an Institute on Digital Humanism, and the Vienna Science and Technology Fund (WWTF) now operates a very successful Digital Humanism research program.

In addition, we succeeded in creating an intellectual core, initially consisting of the authors of Manifesto and the members of our international program committee, and now constantly growing. With the beginning of the pandemic we moved online and organized an online workshop in May 2020 on “Digital Humanism: Informatics in Times of Covid-19”. Since then we run a regular online *Lecture Series*, nearly every second Tuesday (up to now 16 panels and talks, and two workshops), with a growing number of participants, internationally renowned speakers and with topics ranging from AI and ethics, Corona apps and privacy, to the issue of sovereignty in the digital world.^12^ In addition, we currently work on a joint publication and an interdisciplinary Digital Humanism Master program.

## A look into the future

I started this contribution with a personal look backward into the short history of electronic markets, I will conclude with a look into the future. This outlook is obviously highly subjective, and I want to underline, that am not a futurologist^13^. I will name some probable technological and market developments, both being highly interrelated.

- With Internet of Things (IoT), the Internet will be everywhere at any time, and it will be “disappearing”. This is further step into Marc Weiser’s long-lasting vision of ubiquitous computing of the late 80s (Weiser, 1991). This will need new interaction paradigms between humans and computers such as new search and highly personalized interaction approaches, e.g., sensor based, emotional, pro-active, and implicit – we will not even see the “device” or be aware that we are interacting.
- Software will increasingly control and monitor all technical systems. In addition, with simpler technical interfacing system integration will become easier, eventually resulting into real seamless global systems. System as well as software development will more and more benefit from the reuse of existing software from (public) repositories, leading to an evolutionary development process. However, big scale system engineering and software verification will remain a challenge.
- We will see an ongoing interplay of decentralization vs. centralization, take the process from main frame to decentral client-server architectures, then to cloud computing, and now, since it is sometimes obviously essential, edge and fog computing.
- With respect to the increased use of AI techniques, we can expect an integration of data driven approaches with top down logic-based ones, providing semantics and explanation to current systems. However, I am skeptical with respect to strong AI.
- We can expect increasing legal, economic and political pressure and activities against further market concentration as strong network effects will prevail with related economic or political dependency structures. This poses a threat to national sovereignty – governments will react (hopefully).
- I assume that all companies will become – at least partly and contrary to the complaints of traditional players at the ENTER conference in 1994 – “online” companies. They will use IS for internal organization as well as for outside communications, sales and networking. Companies need to pay attention at network engineering, not process engineering.
- Ownership of data in order to understand markets, users / consumers and partners / competitors will be essential, as is the know how to transform data into knowledge.
- Privacy (even more than security) will become the hottest and most controversial issue in the cyber world. Companies, even the big platforms, will not only object, but see this as a business opportunity.
- Competition between different electronic players as well as more global systems (on the same technical infrastructure) will lead to blurring boundaries and easier switching between the different segments. Innovation will continue to come from “outside”, with further waves of new services and an ongoing commoditization of existing services, easing their reuse by others. This will favor networking approaches.

I see the Web, as our world-wide socio-technical system, in a dialectic process or evolution between unorder (permanent innovation with new services and players) and order (ongoing trend to concentration). This is also reflected in my previous list of future technological and market developments, with continuing »laws« of networks and innovation. Understanding this dynamic and even surfing these, often contradicting, waves will need a lot of know-how and intellectual resources. Not surprisingly, this is also where the big IT platform companies are investing. In general, given this complex development and the plethora of technologies, companies will need to play in a clever way the game of cooperation / networking vs. competition, i.e., coopetition, a well-known concept in the IS community.

All this raises content related and institutional challenges for academic teaching and research. Whereas we see already many departments including ethics into their CS and IS curriculum, this trend will be reinforced. But it is not ethics alone, students and University teachers will need to grasp and integrate concepts of other disciplines, from social science and humanities. Take as an example the notion of fairness: it is of central concern in recommender systems and search. But the definition of fairness, or to whom to be fair (individual or group), is not a technical issue, it needs a deeper understanding of, e.g., political science. As Informatics moved into the center of the overall development, we also have to learn that it cannot solve every problem, there are limits of technology. The central position of our discipline also requires humility and a sense of responsibility, it comes along with new challenges, may be bigger than those of the past.

## Conclusions

The development of the Web, facilitating and even driving the digital transformation, is an empirical prove of the *Electronic Markets* approach that technology alone is not sufficient to explain the adoption of technology and to engineer Information Systems. As outlined, it needs a mix of different disciplines as well as technological, organizational, societal, and political perspectives. Interestingly, this is in accordance with the Digital Humanism initiative, which follows a cross-disciplinary and ethical point of view. It has even moved to the political level, as at the end, IT induced economic and societal change is a political question. This is also acknowledged by the President of the European Council Charles Michel (Oct 2, 2020): *“Between the American model of ‘business above all’ and the Chinese state-controlling authoritarian model, there is plenty of room for an attractive and human-centered model*”. Thus, Digital Humanism may also serve as a distinctively European model of digitalization, yet it has a world-wide supporting community.

IT will not stop, nor will the changes it induces. Many of these changes raise and will continue to raise the question of the role of the human in this complex and dynamic man-machine interplay. Most importantly, one to the major statements of the Vienna Manifesto is that we should not only analyze and discuss, but we should also act - both in practical and scientific terms. We are at a crossroads, and the outcome will depend on us, as scientists and citizens; there is nobody else.

## Footnotes

1 https://sdgs.un.org/goals

2 He describes how they started as a kind of rebels, and how they created their own world. As he states they “have disrupted absolutely everything. Politics, finance, education, media, relationships — family relationships, romantic relationships”. But they have– as he continues - no sense of modesty or graciousness. And, even more extreme, “we turned into assholes”.

3 As a side remark: you can see this “winners take it all” mechanism also in “our” increasingly concentrated scientific publication market, e.g., the role of Elsevier as scientific publisher as described by (Klein & Hüllmann, 2018).

4 European countries followed different IT based approaches for their respective Corona contact tracing Apps. At the end it was decided by the two big App OS providers Apple and Google, which alternative to follow. UK and France had to abandon their official national centralized approaches since the two companies did not support it. For Europe, the simple provocative question becomes vital: European sovereignty or digital colony?

5 https://dighum.ec.tuwien.ac.at/

6 There was one very outstanding result: the Manifesto was featured in the Greek newspaper Kathimerini, and this article was then selected as key text for the Greek-wide university entry exams.

7 https://hai.stanford.edu/

8 https://www.humanetech.com/

9 https://www.thedigitalsociety.info/

10 https://digitalenlightenment.org/

11 A detailed discussion of these different initiatives is beyond the scope of this paper.

12 https://dighum.ec.tuwien.ac.at/lectures-program/ and https://www.youtube.com/channel/UC-oCPW9l7IuDvu_J30tqMVw

13 In a marketing note of an innovation and technology conference, the organizers even want to provide a look beyond the future – good luck for such a nonsense!

## References

[CR1] Alt, R., & Klein, S. (2011). Twenty years of electronic markets research—looking backwards towards the future. *Electronic Markets,**21*(1), 41–51. 10.1007/s12525-011-0057-z

[CR2] Clemons, E., & Madhani, N. (2010). Regulation of Digital Businesses with Natural Monopolies or Third-Party Payment Business Models: Antitrust Lessons from the Analysis of Google. *Journal of Management Information Systems, 27*(3), 43–80. 10.2753/MIS0742-1222270303

[CR3] Copeland, D., & McKenney, J. (1988). Airline Reservations Systems: Lessons from History. *MIS Quarterly, 12*(3), 353–370. 10.2307/249202

[CR4] Cusumano M. (2019). “Platformizing' a bad business does not make it a good business. *Communications of the ACM, 63*(1), 23–25. 10.1145/3372918

[CR5] Gawer, A., & Cusumano M. (2013). Industry Platforms and Ecosystem Innovation. *Journal of Product Innovation Management, 31*(3), 417–433. 10.1111/jpim.12105

[CR6] Hardin, G. (1968). The Tragedy of the Commons: The population problem has no technical solution; it requires a fundamental extension in morality. *Science, 162*(3859), 1243–1248. 10.1126/science.162.3859.1243 5699198

[CR7] Kagermann, H., & Wilhelm U. (Eds.) (2020). *European Public Sphere. Towards Digital Sovereignty for Europe*. German National Academy of Science and Engineering. Acatech

[CR8] Klein, S., & Hüllmann, J. (2018). Datenkapitalismus akademischer Wissenschaftsverlage (in German). *Wirtschaftsdienst, 98*(7), 477–480. 10.1007/s10273-018-2318-3

[CR9] Larus, J. , Hankin, C., Carson, S. G., Christen M., Crafa S., Grau, O., Kirchner, C., Knowles, B., McGettrick, V., Tamburri, D. A., & Werthner, H. (2018). *When Computers Decide: European Recommendations on Machine-Learned Automated Decision Making*. Joint report Informatics Europe & EUACM. https://www.informatics-europe.org/publications

[CR10] Lee, E. A. (2020). *The Coevolution. The Entwined Futures of Humans and Machines*. MIT Press.

[CR11] Nida-Rümelin, J., & Weidenfeld, N. (2018). Digitaler Humanismus. *Piper, 2018*.

[CR12] Nygaard, K. (1986). Program Development as a Social Activity In: Kugler, H.J. (ed.) *Information Processing 86*, Elsevier Science, IFIP. *Proceedings from the IFIP 10th World Computer Congress, Dublin, Ireland, pp. 189-198.*

[CR13] Parker, G.G., Alstyne, M.W., & Choudary, S.P. (2016). *Platform Revolution: How Networked Markets Are Transforming the Economy and How to Make Them Work for You*. New York, Norton & Company.

[CR14] Parker, G.G. (2019). *The Transformative Powers of Platform Economies*. https://owncloud.tuwien.ac.at/index.php/s/zlHdqh5Ljue4VhU

[CR15] Popper, K. (1969). Moral Responsibility of the Scientist. *Encounter*. 10.1177/096701067100200311

[CR16] Stark, D., & Pais, I. (2021). Algorithmic Management in the Platform Economy. *Sociologica, International Journal for Sociological Debate 14*(3), 47–72. 10.6092/issn.1971-8853/12221

[CR17] Tedre, M., & Denning, P. (2015). In H. In Werthner & F. van Harmelen (Eds.), *Shifting Identities in Computing: From a useful Tool to a New Method and Theory of Science*. Informatics in the Future. Springer.

[CR18] Vardi, M. (2019). How the Hippies Destroyed the Internet. *Communications of the ACM, 61*(7), 9. 10.1145/3226073

[CR19] Venkatraman, N. (1994). IT-enabled business transformation: from automation to business scope redefinition. *Sloan Management Review, 35*, 73–73.

[CR20] Weiser, M. (1991): The Computer for the 21st Century. *Scientific American, 265*(3), 94–105.

[CR21] Werthner, H. (2001). Just Business – Shouldn’t We Have Some Fun? Invited Paper. *Proceedings of the Electronic Commerce and Web Technologies, ECWEB – DEXA*, Springer Verlag. 10.1007/3-540-44700-8_1

[CR22] Werthner, H. (2020): The Vienna Manifest on Digital Humanism. In Hengstschläger, M / Austrian Council for Research and Technology Development, (Eds.): *Digital Transformation and Ethics*. Salzburg, München: Ecowin Verlag, pp. 338–357.

[CR23] Williamson, O. (1985). *The Economic Institutions of Capitalism*. Macmillan.

[CR24] Zuboff, S. (2019). *The Age of Surveillance Capitalism. The Fight for a Human Future at the New Frontier of Power*. New York, Public Affairs.

